# A Cross-Sectional Dual-Site Analysis of the Gastric Antral and Duodenal Mucosa-Associated Microbiome Across Gastroesophageal Reflux Disease Phenotypes

**DOI:** 10.3390/biomedicines14061221

**Published:** 2026-05-28

**Authors:** Selva Rosyta Dewi, Takashi Matsumoto, Titong Sugihartono, Muhammad Miftahussurur, Yoshio Yamaoka

**Affiliations:** 1 Department of Environmental and Preventive Medicine, Faculty of Medicine, Oita University, 1-1 Idaigaoka, Hasama-machi, Yufu 879-5593, Oita, Japan; selvard17@gmail.com (S.R.D.); tmatsumoto9@oita-u.ac.jp (T.M.); 2 Helicobacter Pylori and Microbiota Study Group, Institute of Tropical Disease, Universitas Airlangga, Surabaya 60115, Indonesia; 3 Research Center for Global and Local Infectious Diseases (RCGLID), Oita University, Yufu 879-5593, Oita, Japan; 4 Division of Gastroenterology-Hepatology, Department of Internal Medicine, Faculty of Medicine, Universitas Airlangga, Surabaya 60132, Indonesia; titongsppd@gmail.com; 5 Department of Medicine, Gastroenterology and Hepatology Section, Baylor College of Medicine, Houston, TX 77030, USA

**Keywords:** gastroesophageal reflux disease, gastric antrum, duodenum, mucosa-associated microbiome, symptom severity, 16S rRNA sequencing

## Abstract

**Background/Objectives**: Despite increasing GERD prevalence worldwide, the role of gastroduodenal microbiota in GERD phenotypes and symptom severity remains poorly understood. This study profiled mucosa-associated microbiota from the gastric antrum and duodenum across phenotypes and examined site-specific associations with symptom severity. **Methods**: In this cross-sectional study, forty individuals, including 26 with erosive reflux disease (ERD), 10 with non-erosive reflux disease (NERD), and 4 participants in the endoscopically normal comparator group, underwent 16S rRNA gene sequencing. Community differences were assessed using Bray–Curtis dissimilarity, differential taxa were explored by linear discriminant analysis effect size (LEfSe), and correlations with validated symptom questionnaires were evaluated. **Results**: Microbial community structure differed significantly between the antrum and duodenum, with *Proteobacteria* and *Firmicutes* predominating at both sites. LEfSe suggested enrichment of *Streptococcus, Haemophilus*, and *Enterobacter* in the duodenum, whereas *Sphingobium, Acinetobacter*, and *Aquabacterium* were more abundant in the antrum. The genus *Helicobacter* was relatively enriched in the antrum of ERD samples, whereas *Streptococcus*-dominant signatures were more prominent in the duodenum. Symptom severity showed stronger associations with duodenal taxa, including *Fusobacterium* with odynophagia, early satiety, and globus; *Aquabacterium* with postnatal drip and dyspnea, whereas gastric associations were fewer. **Conclusions**: In this small exploratory cross-sectional cohort, gastroduodenal microbiota exhibited both site-specific and phenotype-associated differences, with phenotype-related microbial variation being more evident in the duodenum than in the antrum. These hypothesis-generating findings highlight the importance of considering both anatomical context and GERD phenotype in upper gastrointestinal host–microbe interactions, and require confirmation in larger, phenotypically well-characterized cohorts.

## 1. Introduction

Gastroesophageal reflux disease (GERD) is a common chronic disorder of the upper gastrointestinal tract, defined by the reflux of gastric contents into the esophagus [1]. Recent meta-analyses estimate that the global prevalence of GERD symptoms is approximately 14.8% [2]. In North America and Europe, GERD affects roughly 10–20% of the population [3,4], and symptom-based prevalence has also increased across Asia over the past two decades [5]. In Indonesia, the burden of GERD has likewise increased, consistent with trends reported in other Asian and Western populations [5,6]. A survey of Indonesian adults reported a high prevalence of symptom-based GERD, underscoring its substantial public health impact and its burden on patients’ quality of life [6,7]. Within the Americas, GERD represents a particularly significant public health concern, with North America recording the highest regional prevalence at approximately 20% and Latin America and the Caribbean approaching 13%; weekly reflux symptoms affect 11.9% to 31.3% of adults in Latin America—figures substantially exceeding estimates from Asia [4,8]. Chronic complications of GERD are clinically significant, encompassing erosive esophagitis, peptic stricture, Barrett’s esophagus, and esophageal adenocarcinoma [9,10]. Furthermore, GERD imposes a substantial burden on health-related quality of life and healthcare utilization, particularly in patients with persistent or refractory symptoms, and is associated with considerable long-term medical costs [11].

GERD has traditionally been explained by mechanical and chemical factors, including lower esophageal sphincter dysfunction, impaired esophageal clearance, hiatal hernia, and excessive gastric acid exposure [12]. A normal endoscopy does not exclude GERD, and symptom severity does not always correlate with the degree of mucosal erosions or pH-monitored acid exposure, underscoring the need for comprehensive reflux monitoring [13]. Despite being a longstanding and effective treatment for GERD, proton pump inhibitors (PPIs) also fail to deliver an adequate response in about 30% of patients [14,15]. These observations indicate that additional factors may contribute to symptom generation and disease heterogeneity. Clinically, GERD may cause not only troublesome daily symptoms such as heartburn and regurgitation, but also extra-esophageal manifestations and impaired quality of life [16,17]. Its pathogenesis has traditionally been attributed to mechanical and chemical factors, including lower esophageal sphincter dysfunction, impaired esophageal clearance, hiatal hernia, and excessive gastric acid exposure [18,19]. Nocturnal symptom burden is particularly consequential, with over half of patients with severe disease reporting significant sleep disruption that adversely affects both physical functioning and psychological well-being [20]. Over the long term, inadequately managed GERD predisposes affected individuals to a progressive continuum of structural and neoplastic complications—encompassing erosive esophagitis, peptic stricture, Barrett’s esophagus, and esophageal adenocarcinoma—each stage carrying incrementally greater morbidity and clinical complexity [10,18].

In this context, interest in the gastrointestinal microbiome has expanded considerably. Emerging evidence suggests that microbial communities in the upper gastrointestinal tract may influence GERD pathogenesis through modulation of mucosal immunity, inflammation, and sensory signaling [21,22]. The upper gastrointestinal tract, particularly the stomach and duodenum, harbors site-specific microbial ecosystems [23,24,25]. As the gastric antrum and duodenum differ in acid exposure, bile exposure, motility, and mucosal function, comparing these two anatomical sites may help identify whether microbiome alterations relevant to GERD are site-dependent rather than uniformly distributed throughout the upper gastrointestinal tract [26,27,28]. These microbial communities may affect upper gut physiology, including motility, gastric acid exposure, and symptom manifestation [29,30]. Alterations in gastric and duodenal microbiota have also been linked to small intestinal bacterial overgrowth (SIBO) [24,31], increased intraluminal gas production, and disrupted bile acid metabolism, mechanisms that could plausibly exacerbate reflux-related symptoms [32]. These mechanisms may be relevant to GERD because mucosal inflammatory signaling, immune dysregulation, and microbially derived metabolites could influence epithelial sensitivity, barrier function, and upper gastrointestinal symptom perception [22,33,34].

Despite growing interest in microbiome–host interactions, the gastroduodenal microbiota in GERD remains insufficiently characterized. In particular, it is unclear whether microbial patterns differ between anatomical locations and how these differences relate to clinical phenotypes such as erosive reflux disease (ERD) and non-erosive reflux disease (NERD). From a clinical perspective, distinguishing site-specific microbial patterns may improve the interpretation of symptom heterogeneity in GERD. In particular, it remains unclear whether symptom-associated microbial signals are more strongly linked to gastric or duodenal mucosal environments. The primary aim of this study was to compare mucosa-associated microbiota between the gastric antrum and duodenum, while secondary analyses explored associations with GERD phenotypes and symptom severity (GERD-Q and FSSG).

## 2. Materials and Methods

### 2.1. Study Population and Sample Collection

This cross-sectional study enrolled adults aged ≥18 years from the gastroenterology clinics of RSUD Dr. Soetomo Hospital, Surabaya, Indonesia, who were scheduled to undergo upper gastrointestinal endoscopy. Symptomatic participants reported typical reflux symptoms, defined as heartburn and/or regurgitation occurring at least twice weekly for a minimum of three months. An endoscopically normal comparator group was also included. Phenotypic classification based on endoscopic findings and GERD-Q scores is described in Section 2.2.

All enrolled participants underwent gastroscopy to evaluate the esophagus, stomach, and duodenum. Mucosal biopsies for microbiome analysis were obtained from the lesser curvature of the gastric antrum, approximately 3 cm proximal to the pyloric ring, and from the descending duodenum. Biopsy specimens were placed in transport medium containing 10% glycerol. For DNA extraction, samples were suspended in 500 μL phosphate-buffered saline and stored at −80 °C until processing. We acknowledge that upper gastrointestinal mucosal biopsy samples are susceptible to contamination from the oral cavity, endoscope channel, reagents, and other sources, particularly in low-biomass microbiome studies; standard precautions were taken to minimize contamination, including the use of dedicated biopsy forceps and sequential sampling protocols.

Clinical *H. pylori* status was assessed by culture and rapid urease testing, and all included participants were negative by these assessments. However, we acknowledge that false-negative results cannot be completely excluded because each method has inherent diagnostic limitations. Because 16S rRNA gene sequencing provides taxonomic classification primarily at the genus level, any *Helicobacter* reads identified in the sequencing data were not interpreted as evidence of clinical *H. pylori* infection.

Participants who had received *H. pylori* eradication therapy or antibiotics, or had used proton pump inhibitors (PPIs) or other medications likely to affect the upper gastrointestinal microbiota within two weeks before endoscopy, were excluded. Additional exclusion criteria included a history of autoimmune disease, cirrhosis, malignancy, pregnancy, active infection, or major endoscopic abnormalities unrelated to the study groups. The study was approved by the Ethics Committee of RSUD Dr. Soetomo, Surabaya (0124/KPEK/1/2021), and by the Ethics Committee of Oita University Faculty of Medicine (#1660, 2021). Written informed consent was obtained from all participants.

### 2.2. Clinical Data Collection and Symptom Questionnaires

Demographic and clinical data, including age and sex, were collected from all participants. Upper endoscopy was performed by experienced gastroenterologists with at least five years of clinical practice. The severity of erosive esophagitis was graded according to the Los Angeles (LA) classification system. Briefly, LA grade A is defined as one or more mucosal breaks measuring 5 mm or less that do not extend between the tops of adjacent mucosal folds; LA grade B as one or more mucosal breaks longer than 5 mm that likewise do not extend between the tops of adjacent folds; LA grade C as mucosal breaks continuous between the tops of two or more mucosal folds but involving less than 75% of the esophageal circumference; and LA grade D as mucosal breaks involving at least 75% of the esophageal circumference [35].

In accordance with the Lyon Consensus 2.0, LA grade B–D esophagitis was considered objective endoscopic evidence of GERD-related erosive disease, whereas LA grade A findings were considered inconclusive for a definitive diagnosis of GERD [36]. Accordingly, participants with LA grade B–D esophagitis were classified as having erosive reflux disease (ERD), regardless of GERD-Q score. Participants with negative endoscopic findings, defined as normal mucosa or LA grade A esophagitis, and a GERD-Q score ≥8 were classified as having non-erosive reflux disease (NERD). Because ambulatory pH monitoring or pH-impedance testing was not performed, the NERD group may have included patients with heterogeneous underlying mechanisms, such as esophageal hypersensitivity or functional heartburn, which should be considered when interpreting phenotype-level findings. Participants with negative endoscopic findings and a GERD-Q score <8 were classified as the endoscopically normal comparator group. Comparator participants were recruited from the same gastroenterology clinic population and were classified as the endoscopically normal comparator group on the basis of negative endoscopic findings and GERD-Q score criteria; they should not be interpreted as healthy population-based controls. These participants underwent upper gastrointestinal endoscopy for clinical indications. Therefore, the comparator group should not be interpreted as healthy population-based controls or a truly asymptomatic reference population, but rather as an endoscopically normal clinical comparator group within the study classification framework.

All participants completed the GERD Questionnaire (GERD-Q) and the Frequency Scale for the Symptoms of GERD (FSSG) to assess symptom burden (Table S1). Both questionnaires had previously been translated into and validated in Bahasa Indonesia [37,38]. A summary of endoscopic findings, GERD-Q scores, final phenotypic classification, and biopsy availability is provided in Table S2.

### 2.3. Microbiome Analysis

#### 2.3.1. DNA Extraction and 16S rRNA Sequencing

Genomic DNA was extracted using the DNeasy Blood & Tissue Kit (QIAGEN, Santa Clarita, CA, USA) and further concentrated using the DNA Clean & Concentrator kit (Zymo Research, Irvine, CA, USA), according to the manufacturers’ instructions. DNA concentration was measured using the Quantus™ Fluorometer (Promega, Madison, WI, USA). The V3–V4 region of the bacterial 16S rRNA gene was amplified using the universal primers 341F (5′-CCTACGGGNGGCWGCAG-3′) and 805R (5′-GACTACHVGGGTATCTAATCC-3′) [39].

Amplicons were purified using Agencourt AMPure XP magnetic beads (Beckman Coulter, Tokyo, Japan), and short non-target fragments (<500 bp) were removed. Library quality was assessed using the Agilent 2100 Bioanalyzer (Agilent Technologies, Palo Alto, CA, USA) with a DNA 7500 chip. Dual indices and sequencing adapters were added using the Nextera XT Index Kit (Illumina Inc., San Diego, CA, USA). The pooled libraries were further validated using the Agilent TapeStation 4150. Libraries (5 pM) were denatured with 0.2 N NaOH and spiked with 10–15% PhiX Control v3 (Illumina Inc., San Diego, CA, USA) to increase sequence diversity. Sequencing was performed on the Illumina MiSeq platform using 2 × 300 bp paired-end reads with the MiSeq Reagent Kit v3 (Illumina Inc., San Diego, CA, USA), according to the manufacturer’s instructions.

#### 2.3.2. Bioinformatics

Demultiplexed paired-end reads were imported into QIIME 2 (version 2023.9) and processed using the DADA2 plugin to generate amplicon sequence variants (ASVs) [40]. The primer and adapter sequences were removed using Cutadapt (version 4.6), followed by quality filtering [41]. DADA2 was used to trim low-quality regions, model error rates, dereplicate reads, infer ASVs, and remove chimeras. Taxonomy was assigned using a Naïve Bayes classifier trained on the target region against the SILVA database (version 138; 99% sequence identity) [42]. Multiple sequence alignment was performed using MAFFT (version 7.520), and a phylogenetic tree was constructed using FastTree (version 2.2) [43,44].

The feature table, taxonomy, phylogenetic tree, and sample metadata were imported into R (version 4.5.1) using the phyloseq package [45]. For diversity analyses, samples were rarefied to a uniform sequencing depth of 1000 reads per sample in order to retain the maximum number of mucosal biopsy samples for comparative analysis, although we recognize that this relatively low threshold may reduce sensitivity for detecting low-abundance taxa. This cutoff was selected as a compromise between sequencing depth and sample retention, since a higher threshold would have substantially reduced the number of samples available for site-specific and paired analyses. Alpha diversity was assessed using ACE, Chao1, Observed richness, Pielou’s evenness, Shannon, and Simpson indices. Comparisons between anatomical sites (antrum vs. duodenum) were performed using paired non-parametric tests when both samples were available from the same individual (Wilcoxon signed-rank test). For site-specific analyses, including participants with only one biopsy site, unpaired comparisons were performed using the Wilcoxon rank-sum test. Comparisons across GERD phenotypes within each anatomical site were similarly performed using the Kruskal–Wallis test.

Beta diversity was evaluated using Bray–Curtis dissimilarity and visualized by principal coordinate analysis (PCoA) [46,47]. Differences in community composition were tested using permutational multivariate analysis of variance (PERMANOVA) with the adonis2 function in the vegan package [48]. Differentially abundant taxa were explored using linear discriminant analysis effect size (LEfSe); taxa with *p* < 0.05 and an LDA score >2.0 were considered discriminant. Associations between genus-level relative abundance and symptom questionnaire scores were evaluated using Spearman’s rank correlation. Visualizations were generated using ggplot2.

### 2.4. Statistical Analysis

Baseline characteristics are presented using descriptive statistics. Continuous variables are presented as mean ± SD, and categorical variables as counts and percentages. Before applying ANOVA and other parametric tests, the distribution of continuous variables was assessed for normality using the Shapiro–Wilk test. Group comparisons for continuous variables were performed using one-way ANOVA for normally distributed data or the Kruskal–Wallis test, as appropriate. Categorical variables were compared using the Chi-square test or Fisher’s exact test, as appropriate.

For microbiome analyses, alpha-diversity comparisons between paired antral and duodenal samples were performed using the Wilcoxon signed-rank test, whereas unpaired comparisons were performed using the Wilcoxon rank-sum test. Beta-diversity differences were assessed using PERMANOVA based on Bray–Curtis dissimilarity. Homogeneity of multivariate dispersion was additionally assessed to evaluate whether significant PERMANOVA results might reflect differences in dispersion rather than centroid location. As the microbiome data were analyzed as relative abundances, the observed differences should be interpreted within a compositional framework and should not be considered equivalent to absolute abundance changes. As a sensitivity analysis, beta-diversity was additionally assessed using Aitchison distance calculated from centered log-ratio (CLR) transformed count data, without rarefaction, to evaluate the robustness of site-level findings under a compositional framework. Differentially abundant taxa were identified using LEfSe with a significance threshold of *p* < 0.05 and an LDA score >2.0.

Associations between genus-level relative abundance and symptom severity scores were assessed using Spearman’s rank correlation. Because a large number of correlations were tested, these analyses were considered exploratory, and the reported *p*-values are nominal (unadjusted). All tests were two-sided, and *p* < 0.05 was considered statistically significant unless otherwise specified. Clinical statistical analyses were performed using SPSS version 26.0 (SPSS Inc., Chicago, IL, USA), and microbiome analyses and visualization were conducted in R as described above.

## 3. Results

In this study, we first examined overall site-specific differences between the gastric antrum and duodenum as the primary analysis, followed by secondary exploratory analyses of GERD phenotypes, symptom correlations, and paired-site comparisons.

### 3.1. Participants Overview

A total of 40 participants were enrolled, including 26 with erosive reflux disease (ERD), 10 with non-erosive reflux disease (NERD), and 4 endoscopically normal comparator subjects. Participant classification and biopsy availability are summarized in Table S2, and baseline characteristics are shown in Table S3. Paired mucosal biopsies from the gastric antrum and duodenum were collected during upper endoscopy. During sequencing library preparation, samples that did not meet the minimum DNA concentration threshold required for library normalization were excluded. As a result, 25 participants contributed complete paired samples for the final paired analysis, whereas participants with only one biopsy site passing quality control were retained for site-specific analyses. Overall, 35 antral biopsies (25 paired and 10 antrum-only) and 30 duodenal biopsies (25 paired and 5 duodenum-only) were included, yielding 65 mucosal biopsy samples in total. Following QIIME2-based quality filtering and DADA2 denoising, all retained samples exceeded the minimum read count threshold of 500 reads and were included in downstream analyses. The antral dataset comprised 35 biopsies (comparator, *n* = 4; ERD, *n* = 21; NERD, *n* = 10), whereas the duodenal dataset comprised 30 biopsies (comparator, *n* = 3; ERD, *n* = 20; NERD, *n* = 7).

The overall cohort included 27 women (67.5%) and 13 men (32.5%), with a mean age of 42.92 ± 13.87 years. No significant differences in baseline demographic or clinical characteristics were observed across GERD phenotypes, including gender (*p* = 0.259), age (*p* = 0.570), marital status (*p* = 0.700), ethnicity (*p* = 0.154), religion (*p* = 0.158), alcohol consumption (*p* = 0.730), smoking status (*p* = 0.482), diabetes mellitus (*p* = 0.343), hypertension (*p* = 0.189), heart disease (*p* = 0.274), and asthma (*p* = 0.675). Complete baseline comparisons across groups are summarized in Table S3.

### 3.2. Site-Specific Variation in Gastric and Duodenal Microbial Communities

**Figure 1:**
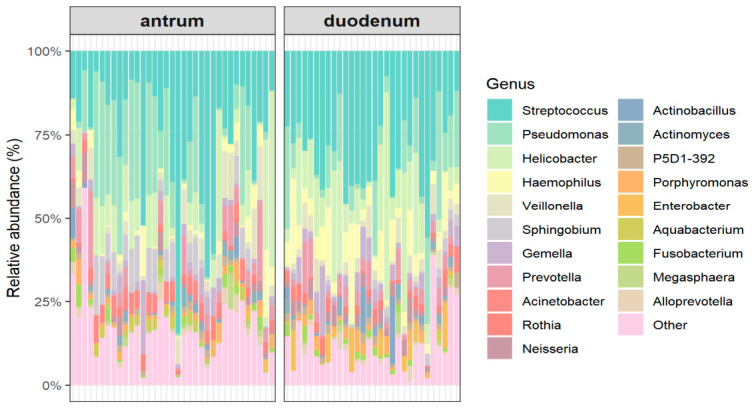
Relative abundance of the top 20 bacterial genera in the gastric antrum and duodenum (*n* = 40 participants, 65 samples).

As the primary overall observation, we first analyzed all retained samples to assess overall site-specific and phenotype-associated patterns. In the overall analysis of all retained samples, a total of 7192 taxa were detected after taxonomic assignment. For downstream diversity analyses, samples were rarefied to a uniform sequencing depth of 1000 reads per sample. This rarefaction depth was selected to maximize sample retention; increasing the threshold to 2000, 3000, or 5000 reads would have resulted in the exclusion of 4, 7, and 16 samples, respectively, representing a disproportionate loss given the small cohort size. At the selected rarefaction depth, taxonomic profiling identified 248 genera belonging to 18 phyla and 153 families in the gastric antrum, and 180 genera belonging to 15 phyla and 115 families in the duodenum. These richness-related observations should be interpreted cautiously because rarefaction to 1000 reads per sample may have reduced sensitivity for detecting low-abundance taxa. The dominant phyla at both sites were *Proteobacteria*, *Firmicutes, Campylobacterota,* and *Bacteroidota*. Phylum-level relative abundance profiles for the antrum and duodenum are shown in Figure S1, and the top 20 genera across the two anatomical sites are presented in Figure 1.

At the genus level, the antrum and duodenum shared several dominant taxa, although their relative abundances differed between sites. In the antrum, the seven most abundant genera were *Streptococcus* (24.3%), *Pseudomonas* (14.6%), *Helicobacter* (9.75%), *Sphingobium* (5.15%), *Prevotella* (4.35%), *Veillonella* (4.07%), and *Haemophilus* (3.14%). In contrast, the duodenum was dominated by *Streptococcus* (29.2%) and *Helicobacter* (13.2%), followed by *Pseudomonas* (9.30%), *Haemophilus* (8.18%), *Gemella* (3.62%), *Prevotella* (3.52%), and *Veillonella* (3.33%). These results indicate that, although several core genera were present at both gastroduodenal sites, their relative dominance was site-specific.

**Figure 2:**
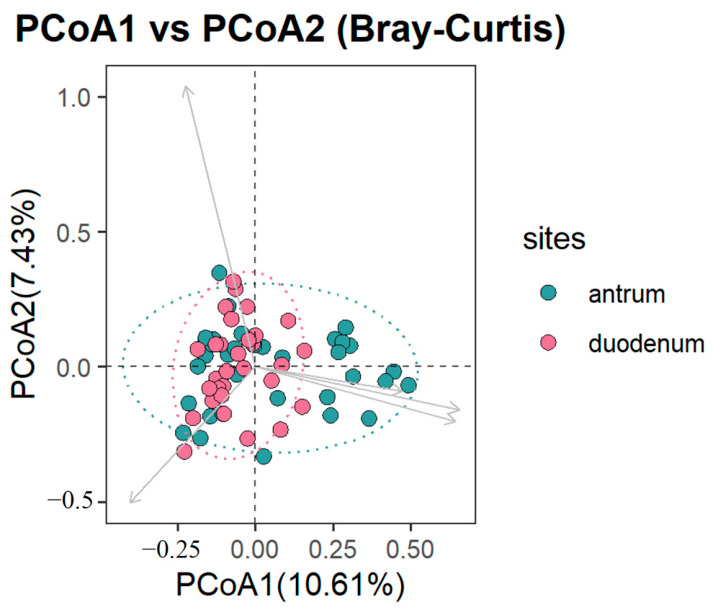
Principal coordinates analysis (PCoA) based on Bray–Curtis distances showing separation of microbial communities between gastric antrum and duodenal samples (PERMANOVA, *p* = 0.018). Antrum samples are shown in green, and duodenal samples are shown in pink (*n* = 40 participants, 65 samples).

To evaluate gastroduodenal mucosa-associated microbial diversity, alpha diversity was assessed using ACE, Chao1, Observed richness, Pielou’s evenness, Shannon, and Simpson indices. No significant differences in alpha diversity were observed between the antrum and duodenum in the alpha diversity analysis (Figure S2). Beta diversity was assessed using Bray–Curtis dissimilarity. Principal coordinate analysis (PCoA) showed significant separation by anatomical site, indicating differences in overall community composition between the antrum and duodenum (PERMANOVA, R^2^ = 0.031, F = 2.037, *p* = 0.018) (Figure 2). Homogeneity of multivariate dispersions was confirmed between sites (betadisper, *p* = 0.448). PERMANOVA findings were interpreted with caution, as significant results may reflect differences in group dispersion as well as differences in community centroid location. As a sensitivity analysis, beta-diversity was reassessed using Aitchison distance on CLR-transformed non-rarefied data; significant site-level separation was maintained (PERMANOVA, R^2^ = 0.022, F = 1.45, *p* = 0.006). To reduce inter-individual variability and more directly compare anatomical sites within the same participant, we next performed a separate paired-sample analysis. A more detailed within-subject paired analysis of site-specific microbiome differences is presented in Section 3.5.

### 3.3. Microbial Differences Across GERD Phenotypes

#### 3.3.1. Microbial Composition Across GERD Phenotypes in the Antrum and Duodenum

As a secondary analysis, phenotype-associated microbial differences were examined across GERD phenotypes (ERD, NERD, and the endoscopically normal comparator group). Relative abundance profiles suggested phenotype-associated shifts in dominant taxa across GERD groups. At the phylum level, a progressive increase in *Proteobacteria* relative abundance was observed across disease groups at both anatomical sites, from the endoscopically normal comparator group (antrum: 42.0%; duodenum: 35.0%) through NERD (antrum: 52.4%; duodenum: 39.7%) to ERD (antrum: 62.1%; duodenum: 51.6%), accompanied by a corresponding progressive decline in *Firmicutes* (endoscopically normal comparator group: antrum 41.2%, duodenum 45.1%; NERD: antrum 22.6%, duodenum 29.8%; ERD: antrum 16.7%, duodenum 22.6%). Notably, *Firmicutes* were the dominant phylum in the duodenum of the endoscopically normal comparator group (45.1%), whereas *Proteobacteria* predominated at both sites in NERD and ERD, as illustrated in Figure S3. These phylum-level findings are descriptive and should be interpreted within the limitations of the small sample size and compositional analysis.

Phenotype-level comparisons, particularly those involving the endoscopically normal comparator group, should be interpreted cautiously because of the limited sample size. In the endoscopically normal comparator group, *Streptococcus* and *Pseudomonas* showed relatively high abundance at both anatomical sites. Descriptively, in NERD, the most abundant genera in the antrum were *Veillonella* (5.68%) and *Pseudomonas* (8.37%), whereas the duodenum was dominated by *Streptococcus* (37.3%) and *Haemophilus* (11.3%).

**Figure 3:**
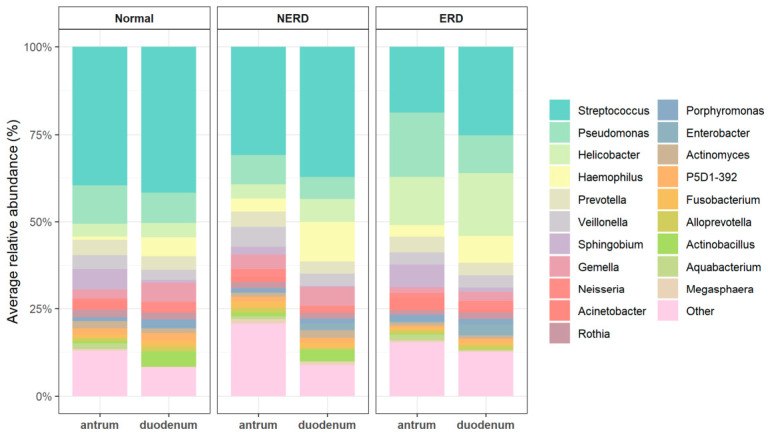
Genus-level microbial composition across GERD phenotypes in the gastric antrum and duodenum (*n* = 40 participants, 65 samples).

**Figure 4:**
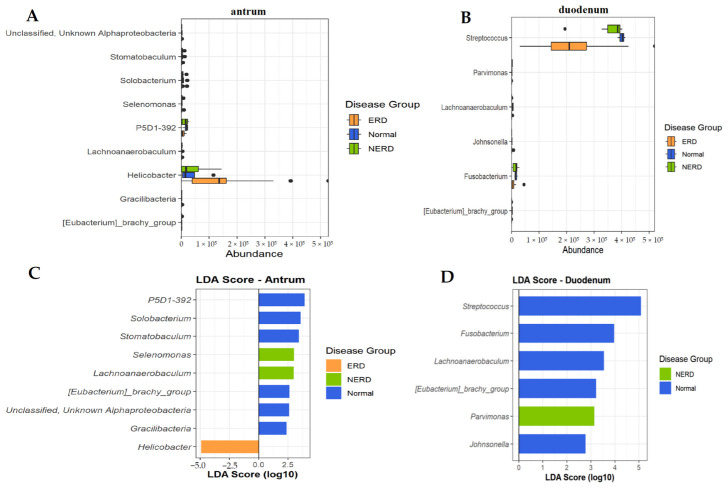
LEfSe analysis showing significantly discriminant bacterial genera across GERD phenotypes within each anatomical site. Relative abundance plots are shown for the gastric antrum (**A**) and duodenum (**B**), and linear discriminant analysis (LDA) score bar plots showing effect sizes are shown for the gastric antrum (**C**) and duodenum (**D**). Taxa with an LDA score >2.0 and *p* < 0.05 were considered discriminant (*n* = 40 participants, 65 samples).

In ERD, the antrum showed higher relative abundance of *Sphingobium* (6.50%), *Acinetobacter* (3.44%), and *Aquabacterium* (1.54%) compared with the duodenum; conversely, the duodenum showed higher relative abundance of *Streptococcus* (25.3%), *Haemophilus* (7.69%), and *Enterobacter* (3.02%) compared with the antrum, as illustrated in Figure 3. Statistical support for differential taxa was assessed using LEfSe; the dominant-genus profiles presented in Figure 4 are descriptive and should be interpreted accordingly.

#### 3.3.2. Alpha and Beta Diversity Differences Across GERD Phenotypes by Anatomical Site

We compared microbial diversity across GERD phenotypes (ERD, NERD, and the endoscopically normal comparator group) separately for antral and duodenal mucosal samples. In the antrum, alpha diversity did not differ significantly among phenotypes based on ACE, Chao1, Observed richness, Pielou’s evenness, Shannon, and Simpson indices, as illustrated in Figure S4A. Similarly, no significant between-group differences in alpha diversity were observed in duodenal samples, as illustrated in Figure S4B.

For beta diversity, microbial community composition in antral samples did not differ significantly among phenotypes (PERMANOVA, R^2^ = 0.085, F = 2.037, *p* = 0.078; Figure S5A), and principal coordinate analysis (PCoA) showed no clear separation by phenotype. In contrast, duodenal samples demonstrated significant differences in beta diversity among phenotypes (PERMANOVA, R^2^ = 0.130, F = 2.012, *p* = 0.003; Figure S5B), with PCoA indicating modest separation of microbial communities across ERD, NERD, and the endoscopically normal comparator group. Homogeneity of multivariate dispersions was confirmed for antral phenotype comparisons (betadisper, *p* = 0.822); however, dispersion differed significantly across phenotype groups in the duodenum (betadisper, *p* = 0.001), indicating that the significant duodenal PERMANOVA result should be interpreted with caution, as it may partly reflect differences in within-group variability rather than differences in community centroid location. These patterns should be interpreted cautiously given the limited sample size, particularly in the endoscopically normal comparator group.

In addition, we performed the sensitivity analysis using CLR-transformed non-rarefied data and Aitchison distance; the pattern of significance differed from the primary analysis: beta-diversity in the antrum site showed significant separation across GERD phenotypes (PERMANOVA, R^2^ = 0.081, F = 1.42, *p* = 0.008), whereas beta-diversity in the duodenum site did not reach statistical significance (PERMANOVA, R^2^ = 0.084, F = 1.24, *p* = 0.082). The inconsistency between the primary and sensitivity analyses likely reflects differences in sensitivity between Bray–Curtis dissimilarity and Aitchison distance, as well as the effect of rarefaction on rare taxa detection, and underscores the exploratory nature of these findings.

#### 3.3.3. Associations Between Microbial Profiles and Symptom Severity Scores

As an exploratory analysis, associations between genus-level microbial relative abundance and symptom severity were assessed using Spearman’s rank correlation. We explored associations between symptom severity and microbial relative abundance at the genus level; because no correction for multiple comparisons was applied across the large number of taxa–symptom pairs tested, this analysis was intended as an exploratory assessment rather than a confirmatory test, and all reported *p*-values are nominal (unadjusted) and should be interpreted with caution. In the primary analysis focusing on the top 20 genera, several nominal associations were observed, as detailed in Table S4.

In the gastric antrum, several dominant genera were significantly associated with reflux-related symptoms. *Pseudomonas* was associated with throat clearing (*p* = 0.013), *Veillonella* with bloating (*p* = 0.014), and *Streptococcus* with postnasal drip (*p* = 0.018) and nausea (*p* = 0.034). *Actinobacillus* in the antrum was associated with multiple symptoms, including regurgitation (*p* = 0.005), belching (*p* = 0.009), and early satiety (*p* = 0.024).

In the duodenum, exploratory correlations were observed between several dominant genera and symptom severity. *Prevotella* showed nominal associations with epigastric pain (*p* = 0.016), throat clearing (*p* = 0.039), and postnasal drip (*p* = 0.035). Additional exploratory correlations were observed between *Veillonella* and early satiety (*p* = 0.018); *Neisseria* and dyspnea (*p* = 0.023); and *Helicobacter* and hoarseness (*p* = 0.025). Notably, *Fusobacterium* in the duodenum was exploratory associated with multiple symptoms, including odynophagia (*p* < 0.001), early satiety (*p* = 0.001), heartburn (*p* = 0.007), and globus sensation (*p* = 0.005). Genus-level associations between microbial taxa and symptoms should be interpreted with caution and do not constitute evidence of a specific biological or causal role of any individual genus in symptom generation.

False discovery rate (FDR) correction was additionally performed as a sensitivity analysis. In the antrum, no associations remained significant after FDR correction. In the duodenum, four associations survived FDR correction: *Fusobacterium* with odynophagia (FDR < 0.001) and early satiety (FDR = 0.020), and *Aquabacterium* with postnasal drip (FDR < 0.001) and dyspnea (FDR = 0.020). These associations should be interpreted cautiously because they derive from exploratory correlational analyses without causal support.

### 3.4. Differential Taxa and Biomarker Identification

LEfSe analysis comparing bacterial taxa between anatomical sites (antrum vs. duodenum) identified multiple taxa with significantly different relative abundances, as detailed in Figure S6A. These phenotype-associated LEfSe findings should be considered exploratory, especially for analyses involving the very small comparator group. Taxa with an LDA score >2.0 and *p* < 0.05 were considered discriminant. At the genus level, *Streptococcus, Haemophilus*, and *Enterobacter* were enriched in the duodenum, whereas *Sphingobium, Acinetobacter*, and *Aquabacterium* were enriched in the antrum, as illustrated in Figure S6B. These findings were consistent with the site-specific relative abundance patterns observed in the descriptive analysis.

To explore phenotype-specific microbial signatures across GERD phenotypes, LEfSe analysis was performed separately for antral and duodenal samples at the genus level. Taxonomic assignment was interpreted at the genus level, and species-level classification of *Helicobacter* was not considered reliable using the present 16S rRNA gene sequencing approach. Accordingly, genus-level *Helicobacter* reads were not interpreted as definitive evidence of active *H. pylori* infection. Taxa were considered significant at *p* < 0.05 with an LDA score >2.0, as illustrated in Figure 4. In the antrum, the genus *Helicobacter* appeared relatively enriched in ERD samples compared with NERD and the endoscopically normal comparator group, although substantial inter-individual variability was observed. Several low-abundance genera, including *Lachnoanaerobaculum*, *P5D1-392, Selenomonas, Solobacterium*, and *Stomatobaculum*, were detected across groups without clear phenotype-specific enrichment. Given the particularly small endoscopically normal comparator group, phenotype-level LEfSe findings should be regarded as exploratory, and LDA scores should be interpreted as indicative of effect magnitude rather than definitive evidence of differential abundance.

In the duodenum, *Streptococcus* was the most abundant genus overall and appeared relatively enriched in ERD and NERD compared with the endoscopically normal comparator group. Other genera, including *Parvimonas, Lachnoanaerobaculum, Johnsonella*, and *Fusobacterium*, were detected at lower relative abundances. Among these, *Fusobacterium* showed greater variability across GERD phenotypes. The observed variability in *Fusobacterium* abundance across phenotypes, while noted descriptively, should be interpreted cautiously given the small group sizes.

### 3.5. Paired Analysis of Gastric and Duodenal Microbiota

**Figure 5:**
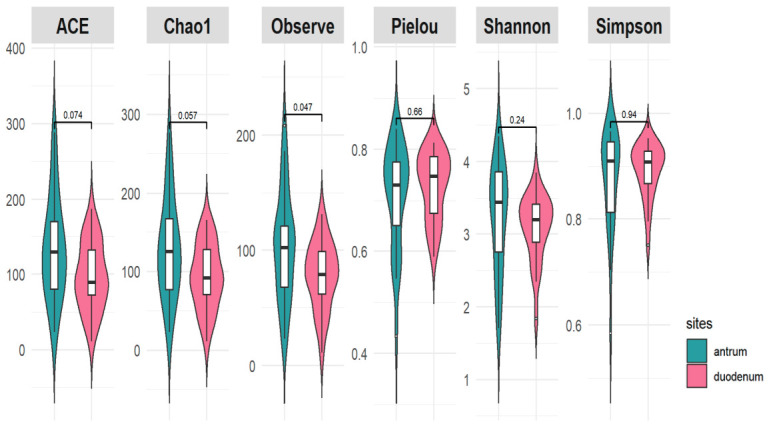
Comparison of α-diversity indices in paired gastric antrum and duodenal samples (*n* = 25 participants, 50 samples).

Paired-sample analyses were conducted as a confirmatory approach to reduce inter-individual variability and strengthen site-level observations. In the paired samples of the antrum and duodenum, alpha-diversity analysis showed that species richness was significantly higher in the antrum than in the duodenum, as indicated by the observed richness index (*p* = 0.047). Although the ACE and Chao1 indices also trended toward higher richness in the antrum, these differences did not reach statistical significance (*p* = 0.074 and *p* = 0.057, respectively). No significant differences were observed in diversity or evenness indices, including Shannon (*p* = 0.24), Pielou’s evenness (*p* = 0.66), and Simpson (*p* = 0.94). These findings suggest that, although species richness differed modestly between sites, overall microbial diversity and evenness were comparable in paired samples, as illustrated in Figure 5.

**Figure 6:**
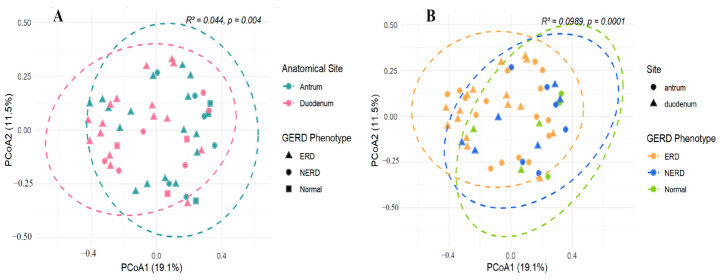
Principal coordinates analysis (PCoA) based on Bray–Curtis distances showing differences in microbial community composition both in (**A**) anatomical site and (**B**) GERD phenotype (*n* = 25 participants, 50 samples).

Beta-diversity analysis incorporating both anatomical site and GERD phenotype revealed that both factors independently explained significant variance in microbial community composition in paired samples; anatomical site explained a modest but significant proportion of variance (PERMANOVA, R^2^ = 0.044, F = 2.37, *p* = 0.004), while disease phenotype explained a greater proportion of variance (PERMANOVA, R^2^ = 0.099, F = 2.66, *p* = 0.0001), as illustrated in Figure 6. Homogeneity of multivariate dispersions was confirmed for all paired-sample beta-diversity comparisons (anatomical site: betadisper, *p* = 0.804; disease: betadisper, *p* = 0.498). These results indicate that the significant PERMANOVA findings reflect differences in community centroid location rather than differences in group dispersion.

As a sensitivity analysis, beta-diversity between paired samples was reassessed using Aitchison distance on CLR-transformed non-rarefied data; significant site-level separation was maintained (PERMANOVA, R^2^ = 0.029, F = 1.443, *p* = 0.003), phenotype (PERMANOVA, R^2^ = 0.070, F = 1.759, *p* = 0.0002), confirming the robustness of this finding independent of rarefaction and normalization approach. In the paired-sample analysis, both anatomical site and disease phenotype independently explained significant variance in microbial community composition when modeled simultaneously.

In the endoscopically normal comparator group, *Streptococcus* was the most dominant genus at both anatomical sites, accounting for 40.1% in the antrum and 41.8% in the duodenum, followed by *Pseudomonas* (14.3% in the antrum and 8.59% in the duodenum). In NERD, *Streptococcus* remained the most abundant genus at both sites (43.4% in the antrum and 39.8% in the duodenum), with *Veillonella* (7.83%) and *Gemella* (6.26%) being the next most abundant genera in the antrum, while *Haemophilus* (11.9%) and *Helicobacter* (7.08%) were prominent in the duodenum. In ERD, *Streptococcus* was also the most abundant genus at both sites (18.8% in the antrum and 26.8% in the duodenum), with *Pseudomonas* (18.6%) and *Helicobacter* (12.8%) being co-dominant in the antrum, while *Helicobacter* (19.2%) and *Pseudomonas* (8.57%) were the next most abundant genera in the duodenum, as illustrated in Figure S7. Genus-level *Helicobacter* reads were consistently detected across both anatomical sites and all disease (58/65 samples, 89.2%), with a mean read count of 589 reads per sample (range 0–4022) and a mean relative abundance of 5.41% in the antrum and 6.61% in the duodenum; however, substantial inter-individual variability was observed, with relative abundance ranging from 0 to 29.8% across samples. Within antral samples, ERD showed the highest mean relative abundance (7.27%; range 0–29.8%) compared with NERD (2.74%; range 0–9.12%) and the endoscopically normal comparator group (2.28%; range 0–7.8%). These reads were not subjected to species-level taxonomic assignment, given the known limitations of 16S rRNA gene sequencing for *Helicobacter* species resolution, and the observed signal should be regarded as a descriptive, genus-level observation only.

LEfSe analysis identified distinct discriminant taxa across GERD phenotypes at both anatomical sites in the paired-sample analysis (LDA score >2.0, *p* < 0.05), as illustrated in Figure S8. In the antrum, all discriminant genera were enriched in the NERD group, including *P5D1-392*, *Oribacterium*, *Solobacterium*, and *Lachnoanaerobaculum*. In the duodenum, *Streptococcus* was the genus with the largest effect size and appeared relatively enriched in the endoscopically normal comparator group, whereas *Lachnoanaerobaculum*, *Solobacterium*, *Parvimonas*, *[Eubacterium]_brachy_group*, and *Johnsonella* were enriched in the NERD group. Notably, no significantly discriminant taxa were identified in the ERD group at either site. In the overall analysis, anatomical site was significantly associated with microbial community composition. When phenotype-level differences were examined by anatomical site, beta-diversity did not differ significantly across GERD phenotypes in the antrum, whereas significant phenotype-associated differences were observed in the duodenum, indicating that phenotype-related microbial variation was more evident in the duodenum than in the antrum. In the paired-sample analysis, both anatomical site and disease phenotype independently explained significant variance in microbial community composition when modeled simultaneously. These phenotype-level findings should be interpreted as exploratory, given the small group sizes, particularly the very small endoscopically normal comparator group, and LDA scores should be regarded as indicative of relative effect magnitude rather than definitive evidence of differential abundance.

## 4. Discussion

GERD is a multifactorial disorder influenced by mechanical, chemical, dietary, and lifestyle-related factors [49,50]. In recent years, the gastrointestinal microbiome has emerged as an additional factor that may contribute to disease heterogeneity and symptom burden [51,52]. In this study, we characterized mucosa-associated microbiota in the gastric antrum and duodenum across GERD phenotypes and examined their associations with symptom severity. In the unpaired analysis, anatomical site was significantly associated with microbial community composition, and phenotype-associated microbial variation was more evident in the duodenum than in the antrum, suggesting that the two anatomical sites may differ in their sensitivity to GERD-related microbial shifts [28,53]. In the paired analysis, both anatomical site and disease phenotype independently contributed to microbial community composition, with phenotype emerging as a comparably influential factor alongside anatomical site. These observations are compatible with both site-specific and phenotype-associated variation in the upper gastrointestinal microbiota, but should not be interpreted as definitive evidence of distinct functional microbial niches, particularly because comparator-group analyses were based on a very small number of samples. We acknowledge that rarefaction to 1000 reads per sample is relatively low for mucosal microbiome analysis and may have reduced sensitivity for robust diversity estimation and detection of low-abundance taxa.

The observed dominance of *Proteobacteria* at both the antrum and duodenum in ERD patients, with a reciprocal relative decline in *Firmicutes*, is consistent with the broader pattern of dysbiosis reported in erosive upper gastrointestinal disease, wherein increased *Proteobacteria* and decreased *Firmicutes* have been described as characteristic features of GERD-associated microbial disruption [22,51,52]. In addition, LEfSe analysis suggested that several discriminant taxa were enriched in NERD, especially in paired analyses, indicating that within-subject comparisons may be particularly useful for detecting subtle phenotype-related microbial signals. These findings support the possibility that NERD is associated with a distinct upper gastrointestinal microbial profile [54,55], although this interpretation remains tentative because the present study lacked objective reflux monitoring, and the non-erosive group may have included patients with esophageal hypersensitivity or functional heartburn [56]. The lack of objective reflux monitoring limits the precision of phenotype assignment and may have reduced the biological interpretability of comparisons involving the NERD group. Furthermore, the endoscopically normal comparator group was particularly small, which limited statistical power and reduced the stability of phenotype-level comparisons and biomarker identification.

The relative enrichment of *Helicobacter* in antral ERD samples should be interpreted cautiously. Although all participants were clinically negative for *H. pylori* by culture and rapid urease testing, the possibility of false-negative results cannot be entirely excluded; accordingly, genus-level *Helicobacter* reads detected by 16S rRNA gene sequencing should not be considered equivalent to confirmed active *H. pylori* infection. This distinction is important because the reported relationship between *H. pylori* and GERD is complex and sometimes paradoxical [39]. Accordingly, genus-level *Helicobacter* associations should be regarded as exploratory observations that may reflect secondary correlational patterns rather than evidence of a direct causal role in symptom generation.

The phenotype-associated enrichment of anaerobic genera in NERD, particularly in the duodenum, is also noteworthy. Taxa such as *Fusobacterium, Solobacterium, Lachnoanaerobaculum,* and *Parvimonas* were more prominent in NERD, whereas *Streptococcus* was enriched in the endoscopically normal comparator group in the paired duodenal analysis. These findings are broadly consistent with observations from the esophageal microbiome, where healthy mucosa is often associated with *Streptococcus*-dominant communities, whereas reflux-related conditions show relative enrichment of Gram-negative or anaerobic taxa [34,57,58]. However, this comparison should be interpreted cautiously, as the present study did not include esophageal samples and therefore cannot directly establish coordinated microbiome shifts across the entire upper gastrointestinal tract. No reagent blanks, endoscope-channel controls, or mock community controls were included in the present study, and this should be considered when interpreting the microbiome findings. Several dominant or discriminant genera identified in this study—including *Streptococcus, Prevotella, Veillonella, Haemophilus, Neisseria, Fusobacterium,* and *Gemella*—are well-established constituents of the healthy oral microbiome [59,60]; the observed site-specific patterns may partly reflect oral or endoscopic carryover rather than exclusively true mucosa-associated microbial differences [61,62]. Accordingly, findings involving these taxa should be interpreted cautiously, and future studies incorporating dedicated contamination controls and decontamination workflows will be necessary to confirm the specificity of the observed upper gastrointestinal microbial signatures.

An additional important observation was that symptom severity showed stronger and more consistent associations with duodenal taxa than with antral taxa. Several duodenal genera were associated with specific symptoms, including *Prevotella* with epigastric pain, and postnasal drip; *Veillonella* with early satiety; *Neisseria* with dyspnea; and *Helicobacter* with hoarseness. In contrast, fewer symptom–taxon associations were observed in the gastric antrum. Although these findings remain exploratory, they raise the possibility that duodenal microbial communities may reflect exploratory symptom-associated patterns that require confirmation in larger studies. Notably, associations involving *Fusobacterium* with odynophagia and early satiety, and *Aquabacterium* with postnasal drip and dyspnea, survived FDR correction, representing the most statistically robust findings of this exploratory analysis. These microbiota–symptom associations should be interpreted as correlational observations and do not establish biological causality. This is biologically plausible because the duodenum functions as an important interface for luminal sensing, neuro-enteric signaling, motility regulation, and exposure to bile and gastric outflow [27,63]. Nonetheless, given the cross-sectional design and small sample size, longitudinal and mechanistic studies will be necessary to determine whether these associations reflect biologically meaningful microbiota–symptom relationships or secondary correlational patterns related to altered gastrointestinal physiology.

Several limitations should be considered when interpreting these findings. First, the sample size was modest, and the endoscopically normal comparator group was particularly small. Second, although biopsies were collected from both sites during endoscopy, not all samples passed library normalization, which reduced the number of complete paired datasets and may have limited statistical power; given that not all collected biopsies were retained, the final analytical dataset may not fully represent the original cohort, and this should be considered when interpreting subgroup and phenotype-level comparisons. Third, objective reflux monitoring, such as ambulatory pHmetry or pH-impedance testing, was not performed, which limited the accuracy of GERD phenotyping. Especially in the non-erosive group, NERD is a heterogeneous clinical category that may include true pathological reflux, reflux hypersensitivity, and functional heartburn, and therefore, the NERD group in the present study should not be interpreted as a biologically homogeneous phenotype. Fourth, dietary intake and some medication-related factors were not systematically assessed, and residual confounding cannot be excluded. Fifth, because biopsies were obtained through a standard endoscope channel, contamination from oral or luminal microbiota is a potential methodological concern. Finally, the use of 16S rRNA gene sequencing limited taxonomic resolution and functional interpretation, and no metabolomic or pathway-based analyses were performed to directly link microbial activity with symptom generation.

Alternative compositional-aware differential abundance methods such as ALDEx2 or ANCOM-BC were not applied in the present study; however, sensitivity analyses using CLR-transformed non-rarefied data and Aitchison distance confirmed the robustness of the primary site-level beta-diversity findings, with significant site-level separation maintained in both all-sample and paired analyses. For phenotype-level comparisons, the CLR sensitivity analysis yielded results that were not fully consistent with the primary Bray–Curtis-based analyses, with the pattern of significance differing between analytical approaches for antral and duodenal samples, likely reflecting differences in sensitivity between distance metrics and the effect of rarefaction on rare taxa detection. Future studies should consider compositional-aware analytical frameworks alongside absolute abundance measurements to further validate the observed microbial patterns. We acknowledge that relative abundance-based analyses, including LEfSe and correlation analyses, are subject to compositional effects and may yield associations that do not necessarily reflect absolute taxon-level changes; accordingly, the reported associations should be interpreted as compositional patterns within this dataset rather than definitive evidence of absolute microbial expansion or reduction. Given the limited sample size and cross-sectional design, these findings should be interpreted as descriptive and hypothesis-generating rather than definitive evidence of clinically meaningful microbiota–symptom relationships.

## 5. Conclusions

These findings indicate site-specific differences in mucosa-associated microbial profiles across GERD phenotypes in this small cross-sectional cohort. The data further suggest that duodenal microbial patterns may warrant further investigation in relation to symptom heterogeneity-associated variation than antral profiles and that paired multi-site sampling improves sensitivity for detecting subtle differences. Phenotype-level analyses, particularly those involving the NERD group, should be explicitly regarded as exploratory given the absence of objective reflux monitoring, the heterogeneous nature of the non-erosive phenotype, and the small comparator group size; these findings should not be interpreted as evidence of biologically distinct NERD-associated microbial signatures. While these findings should be regarded as exploratory, they support the value of anatomical context in upper gastrointestinal microbiome studies and provide a rationale for future work incorporating larger cohorts, objective reflux phenotyping, esophageal sampling, and multi-omics approaches. These observations should be considered descriptive and hypothesis-generating, and no causal or directional inference can be made from the present study.

## Data Availability

All data supporting the findings of this study are included in the article and in the Supplementary Materials. The datasets generated during and/or analyzed during the current study are available from the corresponding author upon reasonable request.

## Supplementary Materials

The following supporting information can be downloaded at: https://www.mdpi.com/article/10.3390/biomedicines14061221/s1, Table S1. Symptom distribution based on GERD-Q and FSSG scores; Table S2. Summary of endoscopic findings and GERD-Q scores; Table S3. Baseline characteristics of study participants; Table S4. Significant correlations between the top 20 bacterial genera and symptom severity in the gastric antrum and duodenum; Figure S1. Stacked bar plot showing phylum-level relative abundances in the gastric antrum and duodenum; Figure S2. Comparison of α-diversity indices in all samples between antrum and duodenum; Figure S3. Relative abundance of dominant bacterial phyla across GERD phenotypes in the gastric antrum and duodenum; Figure S4. Comparison of microbial α-diversity across GERD phenotypes in (A) antral and (B) duodenal samples; Figure S5. Principal coordinates analysis (PCoA) based on Bray–Curtis distances in antrum and duodenum; Figure S6. Taxonomic biomarkers identified by LEfSe analysis between the gastric antrum and duodenum. (A) Relative abundances of discriminative taxa. (B) Linear discriminant analysis (LDA) score bar plot showing effect sizes of discriminative genera (LDA score >2.0, *p* < 0.05); Figure S7. Genus-level microbial composition in paired gastric antrum and duodenal samples across GERD phenotypes; Figure S8. LEfSe analysis of paired samples across GERD phenotypes.

## Abbreviations

Gastroesophageal reflux disease (GERD); Non-erosive reflux disease (NERD); Erosive reflux disease (ERD); GERD questionnaire (GERD-Q); Frequency scale for the symptoms of GERD (FSSG); Body mass index (BMI); Los Angeles classification (LA); Multiple alignment using fast Fourier transform (MAFFT); Amplicon sequence variants (ASVs); Linear discriminant analysis (LDA); Linear discriminant analysis effect size (LEfSe); Principal coordinate analysis (PCoA); 16S ribosomal RNA gene (16S rRNA); Centered log-ratio (CLR) transformed.
