# Supplementary material for: A Cross-Sectional Dual-Site Analysis of the Gastric Antral and Duodenal Mucosa-Associated Microbiome Across Gastroesophageal Reflux Disease Phenotypes
Source: Biomedicines. 2026 May 28;14(6):1221. doi: 10.3390/biomedicines14061221 (PMC13296020; doi:10.3390/biomedicines14061221)
Supplement: Supplementary file 1 [file biomedicines-14-01221-s001.zip › Supplementary Material S1(Tables S1-S4)-SRD.pdf]

Table S1. Symptom distribution based on GERD-Q and FSSG scores.

Subjects	GERD-Q							FSSG												
	1	2	3	4	5	6	Total score	1	2	3	4	5	6	7	8	9	10	11	12	Total Score
**1**	3	3	0	1	3	3	13	4	4	4	3	4	4	4	4	4	4	3	4	46
**2**	3	3	0	1	3	3	13	4	3	3	2	3	4	4	3	2	3	2	4	37
**3**	0	0	0	1	1	0	2	0	0	0	0	0	0	0	0	0	0	3	0	3
**4**	2	2	1	2	2	0	9	2	3	3	1	1	2	2	3	0	1	3	2	23
**5**	3	3	1	1	3	3	14	4	3	4	2	3	4	4	3	2	4	3	4	40
**6**	1	1	0	0	0	2	4	1	3	3	2	0	1	1	3	2	2	2	3	23
**7**	3	2	0	0	3	2	10	4	3	3	2	3	3	3	3	3	2	3	3	35
**8**	1	3	1	0	3	3	11	1	3	3	1	3	1	1	2	1	1	3	1	21
**9**	3	3	0	0	3	0	9	4	4	4	1	4	4	4	3	1	2	4	4	39
**10**	3	0	1	1	3	0	8	0	0	0	0	0	0	0	0	0	0	0	0	0
**11**	3	3	0	0	3	1	10	4	4	4	2	3	4	4	4	1	4	2	4	40
**12**	1	3	1	3	1	2	11	2	3	4	0	0	0	1	4	0	3	4	1	22
**13**	1	1	1	3	1	0	7	1	2	2	1	0	1	1	2	0	1	2	1	14
**14**	0	1	3	3	0	0	7	0	4	4	1	0	0	0	0	0	0	1	0	10
**15**	0	2	3	0	3	0	8	0	2	4	0	0	1	1	4	2	3	3	1	21
**16**	0	2	0	3	0	2	7	1	4	4	1	0	1	1	4	0	4	4	1	25
**17**	0	1	3	3	2	0	9	1	1	0	0	0	0	0	0	0	0	3	0	5
**18**	0	0	2	3	0	0	5	0	0	0	0	0	0	0	0	0	0	1	0	1
**19**	0	3	0	3	3	1	10	0	2	4	0	0	0	0	0	4	4	1	0	15
**20**	0	0	0	0	3	0	3	0	3	2	0	0	2	0	0	0	0	0	0	7
**21**	1	3	1	1	2	0	9	1	3	3	1	0	0	1	3	2	2	0	0	16
**22**	0	0	0	0	0	0	0	0	2	2	0	0	0	0	2	4	0	0	0	10
**23**	3	3	3	1	3	0	13	2	0	0	3	2	1	1	0	1	1	1	1	13
**24**	3	0	0	0	3	3	9	2	0	0	1	1	1	1	0	0	2	0	3	11
**25**	0	0	0	1	3	3	7	0	1	1	1	0	0	0	1	0	0	2	0	6
**26**	0	2	0	2	2	3	9	3	2	2	2	1	3	1	1	0	1	1	4	21
**27**	0	2	0	2	0	3	7	1	2	2	1	3	1	1	2	2	1	1	4	21
**28**	3	3	0	3	3	3	15	4	4	4	2	0	4	4	4	3	4	4	4	41
**29**	2	0	0	3	3	3	11	1	2	1	1	0	1	1	3	1	1	3	1	16
**30**	2	1	0	3	2	3	11	3	1	1	2	1	1	1	1	1	1	1	1	15
**31**	3	3	1	2	1	2	12	3	1	1	2	2	0	2	2	3	2	1	1	20
**32**	3	3	0	0	3	3	12	2	3	3	1	1	1	1	3	1	1	3	3	23
**33**	1	3	0	1	3	3	11	2	3	3	0	3	3	1	2	4	0	4	1	26
**34**	3	3	0	2	0	3	11	3	3	2	2	1	2	1	2	1	1	4	3	25

Table S1. *Continue..*

Subjects	GERD-Q							FSSG												
	**1**	**2**	**3**	**4**	**5**	**6**	**Total score**	**1**	**2**	**3**	**4**	**5**	**6**	**7**	**8**	**9**	**10**	**11**	**12**	**Total Score**
**35**	1	2	2	1	3	3	12	3	3	2	2	3	3	3	2	2	3	3	3	32
**36**	2	0	1	1	3	0	7	0	0	3	0	2	1	0	2	0	2	3	0	13
**37**	2	0	3	3	3	1	12	0	2	2	0	2	0	0	1	2	0	0	0	9
**38**	0	1	3	1	0	3	8	1	1	2	0	0	0	0	2	2	2	3	0	13
**39**	3	0	3	3	3	2	14	4	4	4	0	0	1	1	1	3	0	1	0	19
**40**	2	0	2	3	0	3	10	2	2	3	1	2	1	1	3	0	3	0	0	18

Table S2. Summary of endoscopic findings and GERD-Q scores.

Subjects	Endoscopic/symptom -based classification	GERD-Q score	Category	Biopsy
1	GERD	13	ERD	antrum, duodenum
2	GERD	13	ERD	antrum, duodenum
3	GERD	2	ERD	antrum, duodenum
4	GERD	9	ERD	antrum
5	GERD	14	ERD	antrum
6	GERD	4	ERD	antrum, duodenum
7	GERD	10	ERD	antrum
8	Normal	11	NERD	antrum, duodenum
9	Normal	9	NERD	antrum, duodenum
10	GERD	8	ERD	antrum, duodenum
11	GERD	10	ERD	antrum, duodenum
12	GERD	11	ERD	antrum, duodenum
13	GERD	7	ERD	antrum
14	Normal	7	Normal	antrum, duodenum
15	Normal	8	NERD	antrum, duodenum
16	Normal	7	Normal	antrum
17	Normal	9	NERD	antrum, duodenum
18	Normal	5	Normal	antrum, duodenum
19	Normal	10	NERD	antrum, duodenum
20	Normal	3	Normal	antrum, duodenum
21	GERD	9	ERD	antrum, duodenum
22	GERD	0	ERD	antrum, duodenum
23	NERD	13	NERD	antrum, duodenum
24	NERD	9	NERD	antrum, duodenum
25	GERD	7	ERD	duodenum
26	GERD	9	ERD	antrum, duodenum
27	GERD	7	ERD	antrum, duodenum
28	GERD	15	ERD	antrum, duodenum
29	GERD	11	ERD	antrum, duodenum
30	GERD	11	ERD	antrum, duodenum
31	GERD	12	ERD	duodenum
32	GERD	12	ERD	duodenum
33	GERD	11	ERD	duodenum
34	GERD	11	ERD	antrum, duodenum
35	Normal	12	NERD	antrum
36	GERD	7	ERD	duodenum
37	Normal	12	NERD	antrum
38	GERD	8	ERD	antrum
39	GERD	14	ERD	antrum
40	Normal	10	NERD	antrum

Table S3. Baseline characteristics of study participants.

Characteristics	ERD (n=25)	NERD (n=11)	Normal (n=4)	Full subjects (n=40)	*p*-value
**Gender, n (%)**					
Male	9 (36.0)	4 (36.4)	0 (0)	13 (32.5)	0.259
Female	16 (64.0)	7 (63.6)	4 (100)	27 (67.5)	
**Age (years)**	44.00 ± 14.74	39.20 ± 11.49	48.25 ± 9.57	42.92 ± 13.87	0.570
**Marital status, n (%)**					
Not married	22 (88.0)	7 (63.6)	4 (100)	33 (82.5)	0.700
Married	2 (8.0)	3 (27.3)	0 (0)	5 (12.5)	
Divorce	1 (4.0)	1 (9.1)	0 (0)	2 (5.0)	
**Ethnicity, n (%)**					
Javanese	20 (80.0)	6 (54.5)	3 (75)	29 (72.5)	0.154
Madurase	4 (16.0)	2 (18.2)	1 (25)	7 (17.5)	
Chinese	1 (4.0)	0 (0)	0 (0)	1 (2.5)	
Balinese	0 (0)	3 (27.3)	0 (0)	3 (7.5)	
**Religion, n (%)**					
Islam	23 (92.0)	9 (81.8)	4 (100)	36 (90.0)	0.158
Hinduism	0 (0)	2 (18.2)	0 (0)	2 (5.0)	
Christian	2 (8.0)	0 (0)	0 (0)	2 (5.0)	
**Drinking alcohol, n (%)**					
Yes	2 (8.0)	1 (9.1)	0 (0)	3 (7.5)	0.730
No	20 (80.0)	6 (54.5)	4 (100)	30 (67.5)	
No data	3 (12.0)	4 (36.4)	0 (0)	7 (17.5)	

Table S3. *Continue..*

Characteristics	ERD (n=25)	NERD (n=11)	Normal (n=4)	Full sample (n=40)	*p*-value
**Smoking, n (%)**					
Yes	6 (24.0)	2 (18.2)	0 (0)	8 (20.0)	0.482
No	16 (64.0)	5 (45.4)	4 (100.0)	25 (62.5)	
No data	3 (12.0)	4 (36.4)	0 (0)	7 (17.5)	
**Diabetes mellitus, n (%)**					
Yes	1 (4.0)	0 (0)	1 (25.0)	2 (5.0)	0.343
No	9 (36.0)	5 (45.5)	2 (50.0)	16 (40.0)	
No data	15 (60.0)	6 (54.5)	1 (25.0)	22 (55.0)	
**Hypertension, n (%)**					
Yes	4 (16.0)	0 (0)	0 (0)	4 (10.0)	0.189
No	8 (32.0)	5 (45.5)	3 (75.0)	16 (40.0)	
No data	13 (52.0)	6 (54.5)	1 (25.0)	20 (50.0)	
**Heart disease, n (%)**					
Yes	3 (12.0)	0 (0)	0 (0)	3 (7.5)	0.274
No	8 (32.0)	5 (45.5)	3 (75.0)	16 (40.0)	
No data	14 (56.0)	6 (54.5)	1 (25.0)	21 (52.5)	
**Asthma, n (%)**					
Yes	1 (4.0)	1 (9.1)	0 (0)	2 (5.0)	0.675
No	9 (36.0)	4 (36.4)	3 (75.0)	16 (40.0)	
No data	15 (60.0)	6 (54.5)	1 (25.0)	22 (55.0)	

Table S4. Significant correlations between the top 20 bacterial genera and symptom severity in the gastric antrum and duodenum.

Antrum					Duodenum				
**Symptoms**	**Genus**	**Spearman correlation coefficient (ρ)**	**p-value**	**FDR**	**Symptoms**	**Genus**	**Spearman correlation coefficient (ρ)**	**p-value**	**FDR**
Heartburn	*Enterobacter*	0.334	0.049*	0.564	Heartburn	*Fusobacterium*	-0.484	0.007*	0.140
Regurgitation	*Actinobacillus*	-0.463	0.005**	0.100	Regurgitation	-	-	-	
Bloating	*Veillonella*<br>*Fusobacterium*	-0.410<br>-0.398	0.014*<br>0.018*	0.180<br>0.180	Bloating	-	-	-	
Nausea	*Streptococcus*<br>*Megasphaera*	-0.359<br>-0.372	0.034*<br>0.028*	0.340<br>0.340	Nausea	*Porphyromonas*	-0.415	0.023*	0.460
Epigastric pain	-	-	-		Epigastric pain	*Prevotella*	-0.434	0.016*	0.320
Odynophagia	-	-	-		Odynophagia	*Fusobacterium*	-0.599	<0.001***	<0.001*
Early satiety	*Actinobacillus*	-0.380	0.024*	0.480	Early satiety	*Veillonella*<br>*P5D1-392*<br>*Fusobacterium*	-0.428<br>-0.362<br>-0.594	0.018*<br>0.049*<br>0.001***	0.180<br>0.254<br>0.020*
Globus	*Actinomyces*<br>*P5D1-392*	-0.337<br>-0.404	0.048*<br>0.016*	0.320<br>0.160	Globus	*Fusobacterium*	-0.501	0.005**	0.100
Belching	*Porphyromonas*<br>*Actinobacillus*	-0.348<br>-0.437	0.041*<br>0.009*	0.410<br>0.180	Belching	-	-	-	
Hoarseness	-	-	-		Hoarseness	*Helicobacter*<br>*P5D1-392*	0.408<br>-0.416	0.025*<br>0.022*	0.250<br>0.250
Postnasal drip	*Streptococcus*<br>*Pseudomonas*	-0.359<br>0.334	0.018*<br>0.047*	0.360<br>0.250	Postnasal drip	*Prevotella*<br>*Aquabacterium*<br>*Megasphaera*	0.386<br>0.628<br>0.395	0.035*<br>0.000***<br>0.031*	0.233<br>0.000*<br>0.233
Dyspnea	-	-	-		Dyspnea	*Neisseria*<br>*P5D1-392*<br>*Aquabacterium*	-0.413<br>-0.380<br>0.585	0.023*<br>0.039*<br>0.001***	0.230<br>0.240<br>0.020*

Analyzed using Spearman rank correlation test; * Statistically significant at p<0.05.
