# Supplementary material for: A Cross-Sectional Dual-Site Analysis of the Gastric Antral and Duodenal Mucosa-Associated Microbiome Across Gastroesophageal Reflux Disease Phenotypes
Source: Biomedicines. 2026 May 28;14(6):1221. doi: 10.3390/biomedicines14061221 (PMC13296020; doi:10.3390/biomedicines14061221)
Supplement: Supplementary file 1 [file biomedicines-14-01221-s001.zip › Supplementary Material S2(Figures S1-S8)-SRD.pdf]

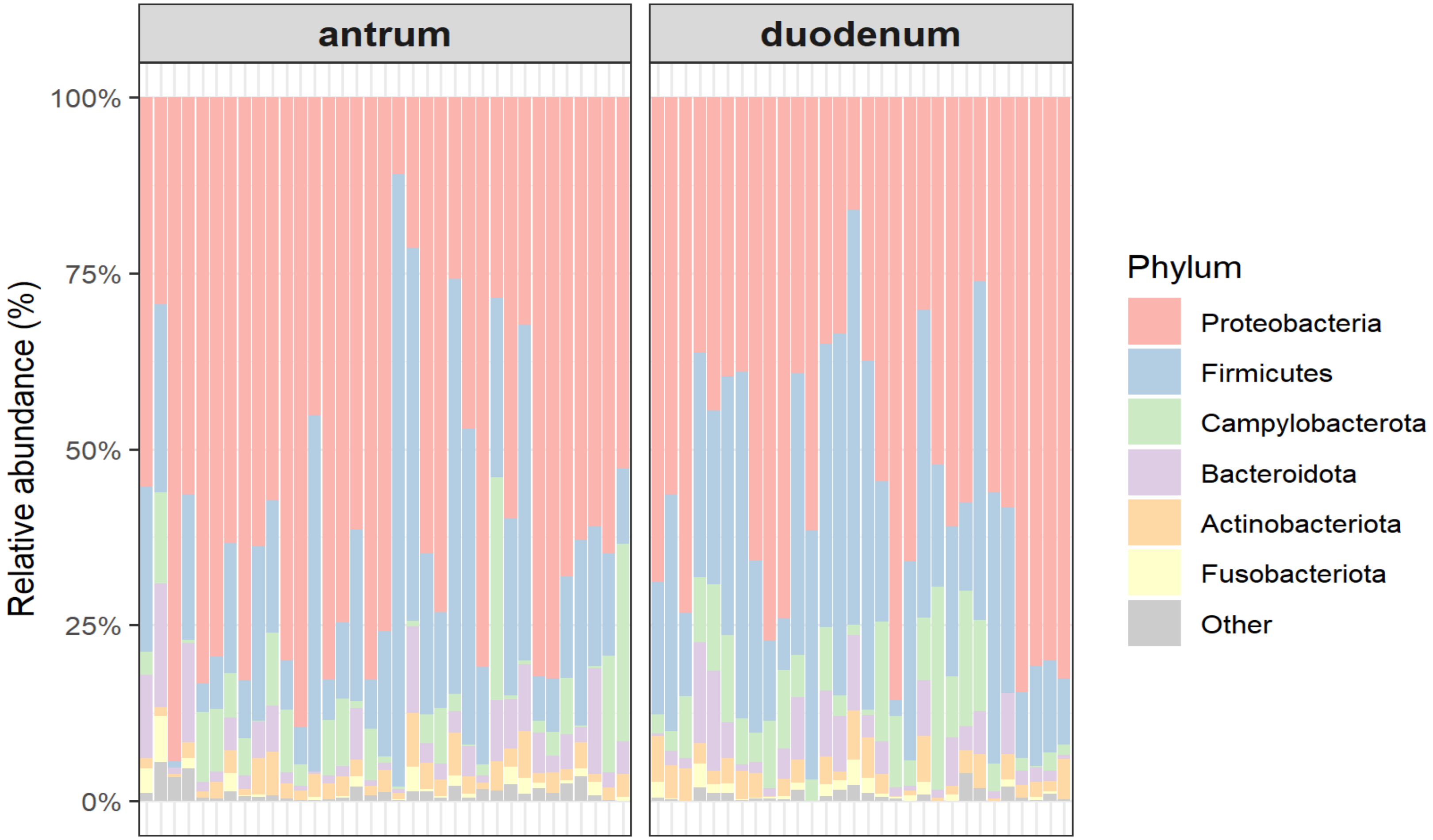

Figure S1. Stacked bar plot showing phylum-level relative abundances in the gastric antrum and duodenum.

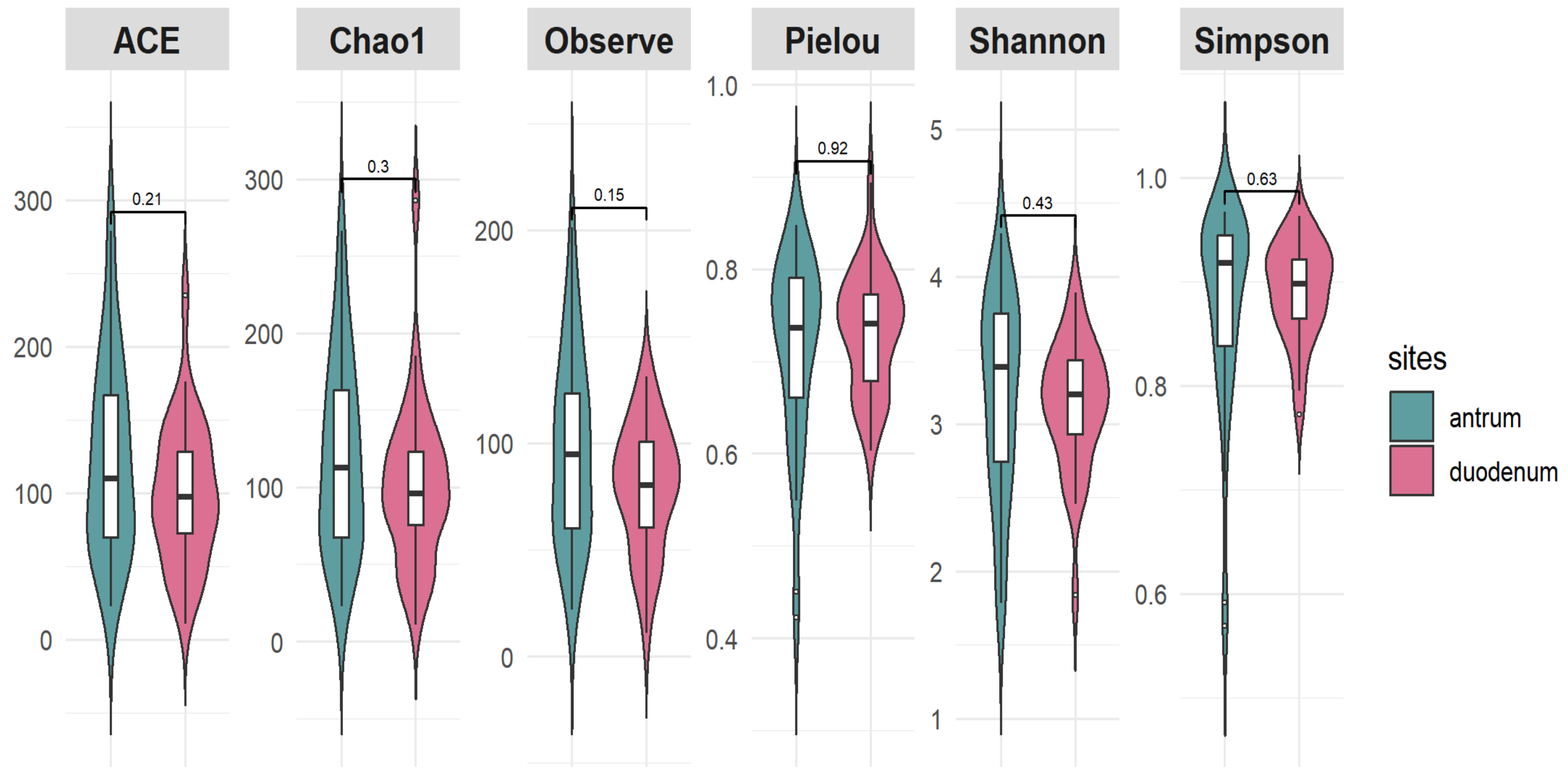

Figure S2. Comparison of α-diversity indices in all samples between antrum and duodenum.

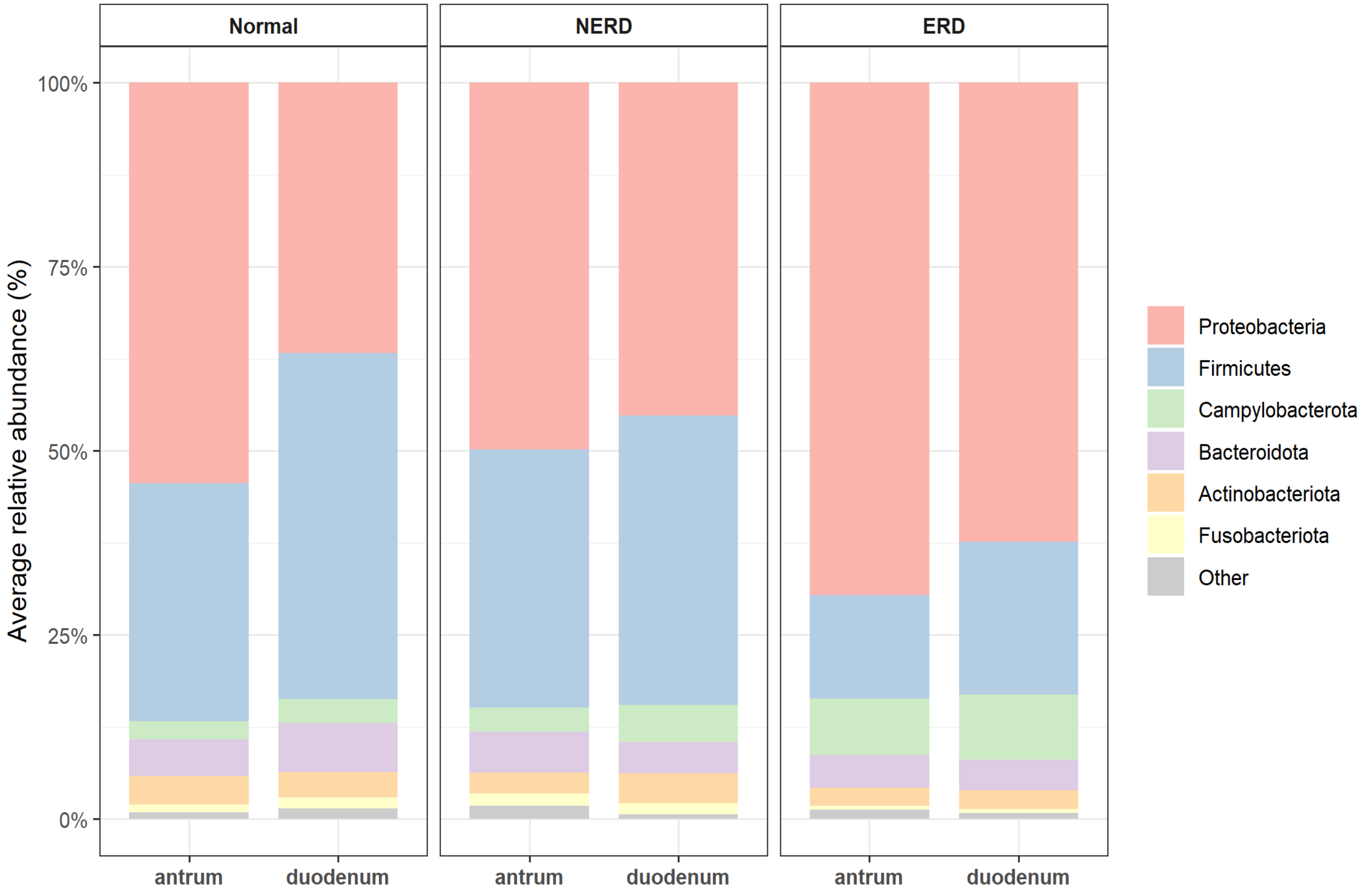

Figure S3. Relative abundance of dominant bacterial phyla across GERD phenotypes in the gastric antrum and duodenum.

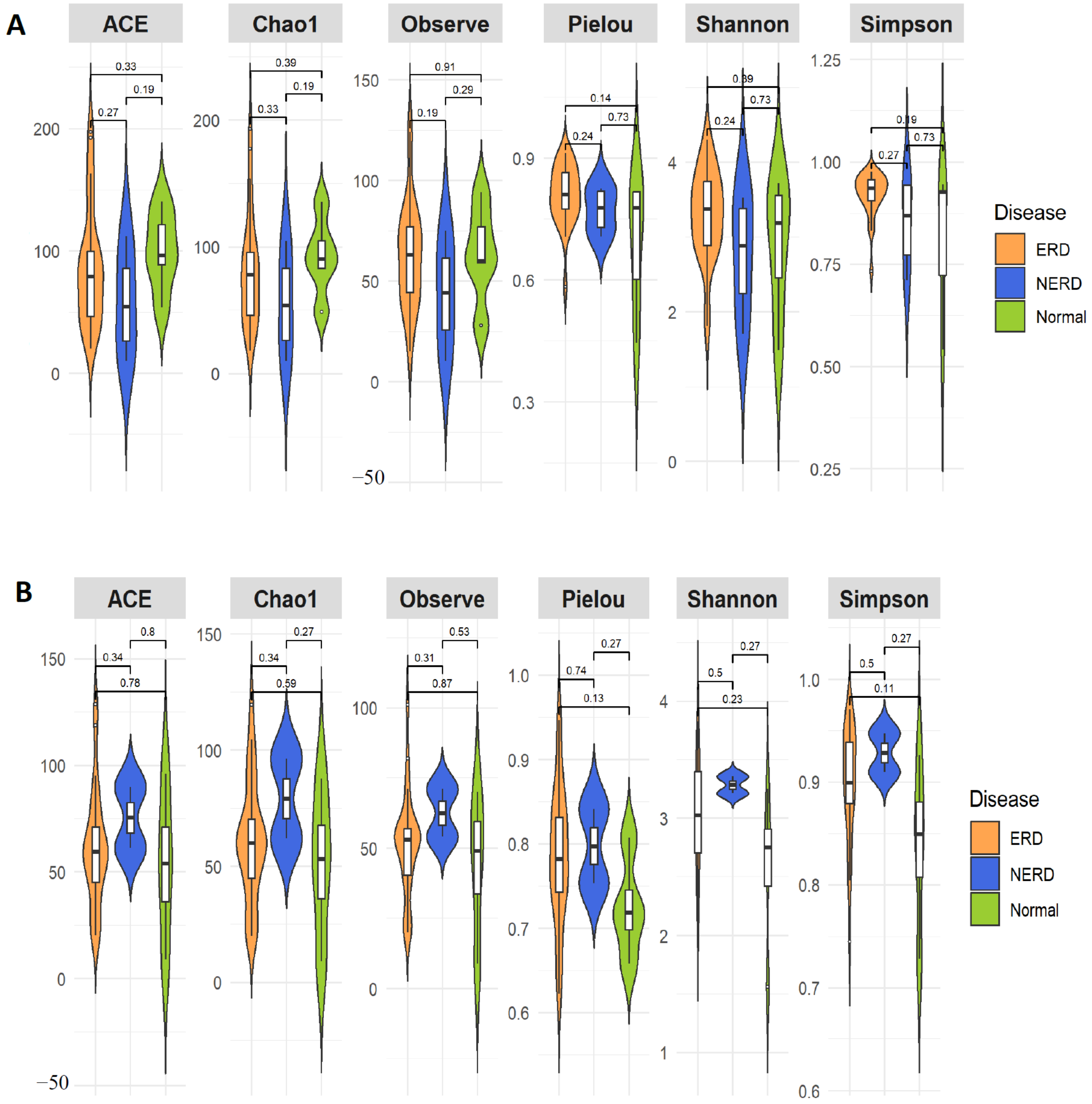

Figure S4. Comparison of microbial α-diversity across GERD phenotypes in (A) antral and (B) duodenal samples.

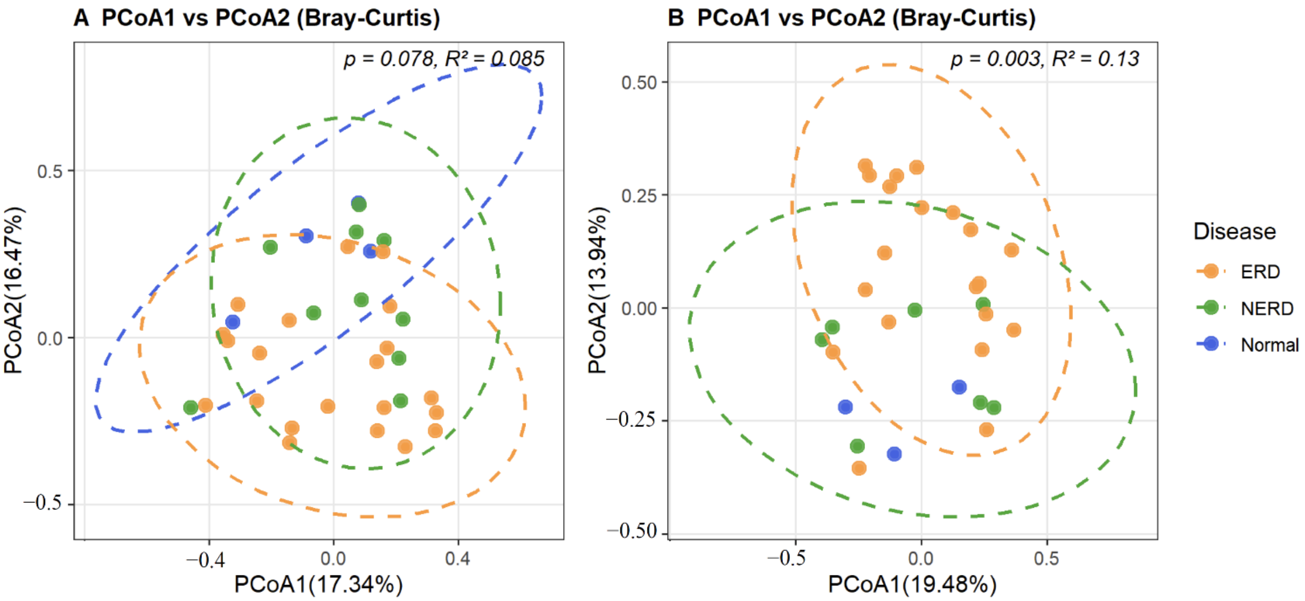

Figure S5. Principal coordinates analysis (PCoA) based on Bray-Curtis distances in (A) antrum ($p = 0.078$) and (B) duodenum ($p = 0.003$).

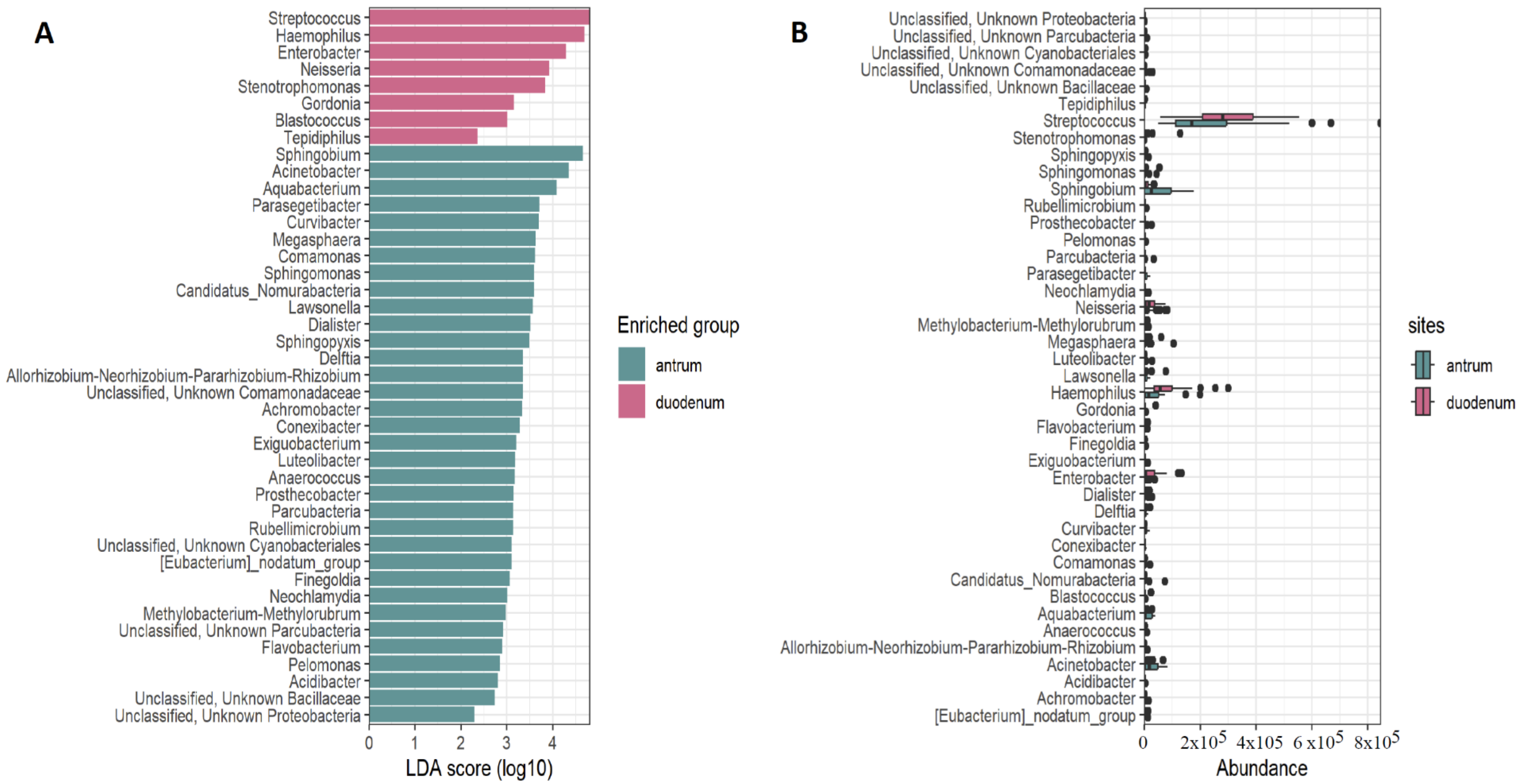

Figure S6. Taxonomic biomarkers identified by LEfSe analysis between the gastric antrum and duodenum. (A) Relative abundances of discriminative taxa. (B) Linear discriminant analysis (LDA) score bar plot showing effect sizes of discriminative genera (LDA score > 2.0, $p < 0.05$).

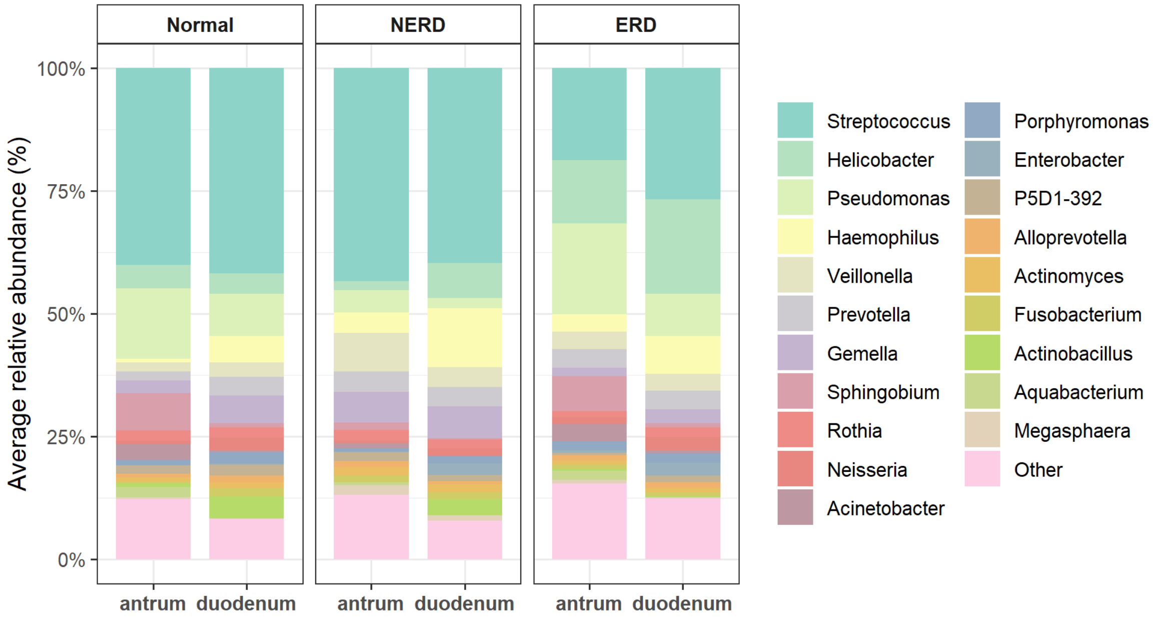

Figure S7. Genus-level microbial composition in paired gastric antrum and duodenal samples across GERD phenotypes.

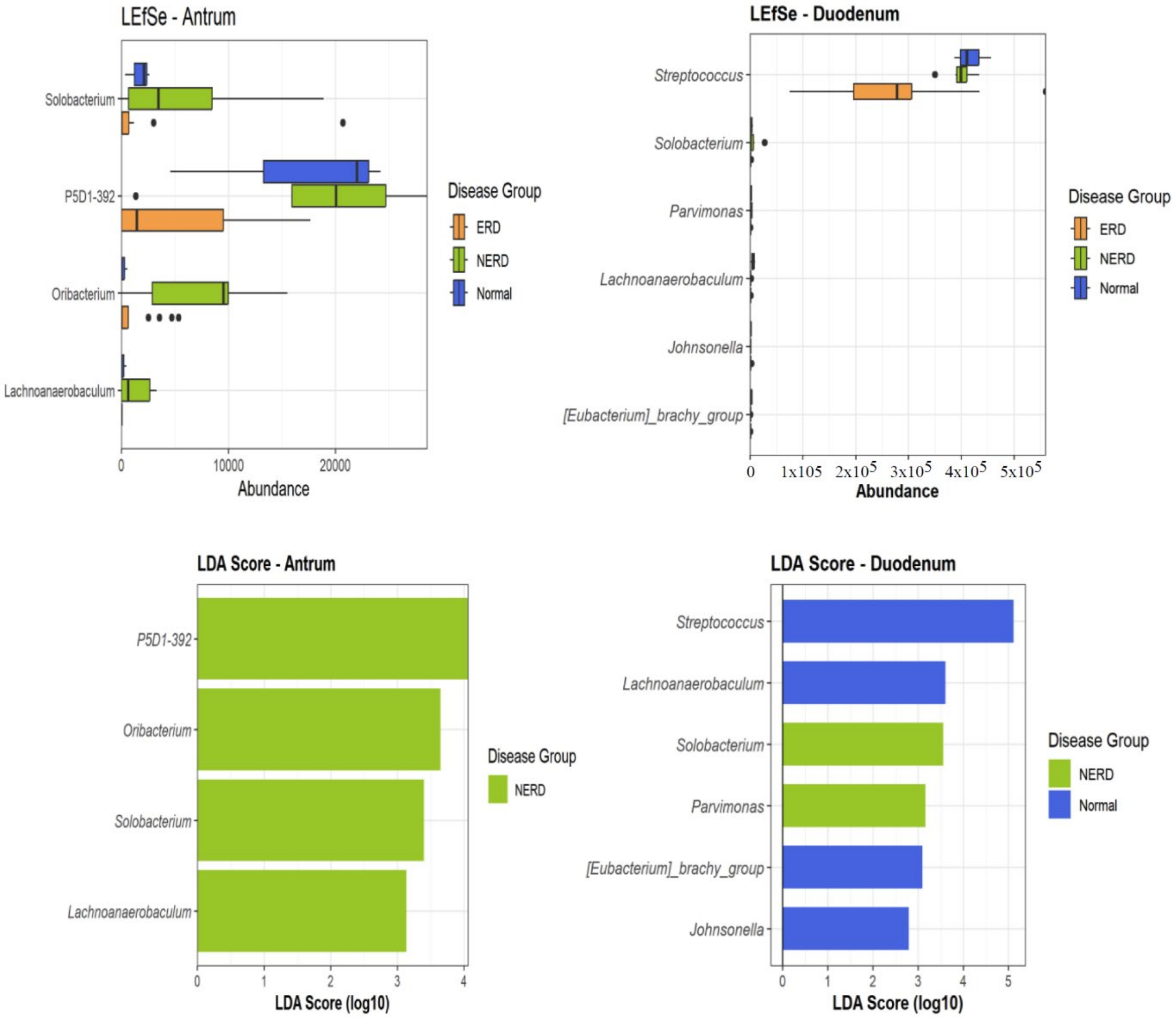

Figure S8. LEfSe analysis of paired samples across GERD phenotypes. Relative abundance plots of discriminative bacterial genera are shown for the gastric antrum (left) and duodenum (right), together with corresponding LDA score bar plots (LDA score > 2.0, $p < 0.05$).
